# Kisspeptin‐10 Prevents the Development of Cerebral Aneurysms by Reducing the Expression of Egr‐1

**DOI:** 10.1111/cns.70413

**Published:** 2025-05-19

**Authors:** Huimin Yu, Minghong Xie, Xuancong Liufu, Yezi Xu, Lei Chen

**Affiliations:** ^1^ Department of Neurology The First Dongguan Affiliated Hospital of Guangdong Medical University Dongguan City Guangdong Province China; ^2^ Department of Neurosurgery The First Dongguan Affiliated Hospital of Guangdong Medical University Dongguan City Guangdong Province China; ^3^ Department of Neurosurgery Dongguan Kanghua Hospital Dongguan City Guangdong Province China

**Keywords:** angiogenesisangiogenesis, cerebral aneurysms, Egr‐1, Gpr54, Kisspeptin‐10

## Abstract

**Aims:**

Cerebral aneurysms (CAs) are a prevalent brain condition with poorly understood pathological features. The Kisspeptin‐10 (KP‐10)/G protein‐coupled receptor 54 (GPR54) system is a vital neuroendocrine pathway primarily implicated in the regulation of reproductive functions and energy metabolism. This research explores the role of the KP‐10/GPR54 system in CAs.

**Materials and Methods:**

Serum levels of KP‐10 in CA patients and animal models were assessed using commercial ELISA kits. Mice were divided into five groups: WT, GPR54^−/−^, CA, CA + KP‐10, and CA + GPR54^−/−^ + KP‐10. The CA profiles were evaluated using Verhoeff‐van Gieson staining. Human brain microvascular endothelial cells (HBMVECs) were stimulated with Ang II (10^−7^ mol/L) with or without KP‐10 (50, 100 nM). Angiogenic tube formation was then assessed.

**Results:**

We found that KP‐10 levels were reduced in both CA patients and mouse models. In CA mice, Gpr54 expression in the Circle of Willis (COW) was also decreased. KP‐10 reduced CA size in wild‐type mice, but not in Gpr54 knockout mice. It also reduced matrix metalloproteinase‐9 (MMP‐9), macrophage infiltration, and vascular endothelial growth factor‐A (VEGF‐A) expression, effects that were absent in Gpr54 knockout mice. In vitro, KP‐10 inhibited Ang II‐induced proliferation, angiogenic tube formation, and VEGF‐A expression in HBMVECs by reducing early growth response‐1 (Egr‐1). These effects were abolished when Gpr54 was knocked down, indicating that KP‐10's action is dependent on Gpr54.

**Conclusion:**

This study shows that KP‐10 binding to Gpr54 inhibits Egr‐1 expression, thereby suppressing MMP‐9 and VEGF‐A, reducing macrophage infiltration and angiogenesis, and preventing cerebral aneurysm development. Thus, the KP‐10/Gpr54 system is a key therapeutic target for the treatment of cerebral aneurysms.

## Graphical abstract

The Kisspeptin‐10 (KP‐10)/GPR54 signaling pathway is dysregulated in both individuals with cerebral aneurysms and experimentally induced mice. KP‐10 administration prevents CA formation in wild type but not GPR54 KO mice, highlighting the crucial role of GPR54 in mediating KP‐10's protective effects. KP‐10 inhibits the expression of MMP‐9 and VEGF‐A in the COW region of CA mice, indicating its potential to suppress angiogenesis and aneurysm growth. KP‐10 treatment ameliorates Ang II‐induced cell proliferation and tube formation in HBMVECs by inhibiting Egr‐1 expression, with overexpression of Egr‐1 abrogating these beneficial effects.

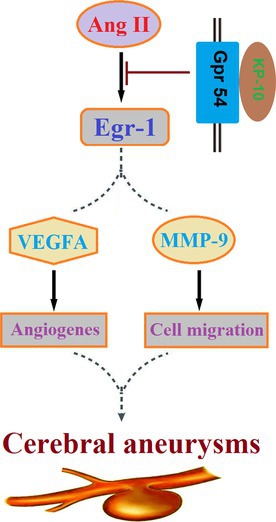

## 1 Introduction

Cerebral aneurysms (CAs) are intracranial vascular lesions that occur due to the focal dilation of the arterial wall, leading to the formation of a vascular aneurysmal protrusion. They are most commonly found at the branching points of the cerebral arteries, particularly in the Circle of Willis (COW), with the anterior half of the Willis circle being the most frequent site, accounting for approximately 85% of all occurrences [1]. CAs pose a severe threat to human life, have a high incidence of rupture leading to cerebrovascular accidents, and are the most direct cause of spontaneous subarachnoid hemorrhage. The risk of a cerebrovascular accident is third only to hypertension and cerebral thrombosis [2]. CAs occur at any age, but patients are often middle‐aged and elderly women between 40 and 60 years old. As clinical understanding of the dangers of CA deepens, related research is increasing. Many epidemiologists have studied and analyzed the disease, suggesting that its occurrence is influenced by multiple factors, including smoking, alcohol consumption, hypertension, and diabetes, all of which are risk factors [3]. However, the pathogenesis of CA is not well understood, and there is no clear morphological analysis of the aneurysm. Elucidating the pathogenesis of CA and seeking corresponding treatment strategies are of significant clinical importance.

Brain microvascular endothelial cells (BMVECs) fulfill a crucial function in CA developments. BMVECs not only respond to changes in hemodynamics, such as wall shear stress (WSS) and WSS gradient (WSSG), but also participate in the inflammatory response and vascular remodeling during aneurysm formation and rupture. High WSS and WSSG are related to vascular remodeling and the eventual formation of aneurysms [4]. At the site of aneurysm formation, ECs are damaged by the impact of blood flow, allowing inflammatory cells and substances to more easily enter the subintima, causing inflammation and further degradation of the vascular wall [5]. Additionally, functional changes in ECs, such as the release of nitric oxide, are also related to aneurysm formation. Damage and dysfunction of ECs can lead to the degradation and weakening of the vascular wall, thereby promoting the growth and rupture of aneurysms [6]. Overexpression of matrix metalloproteinases (MMPs) on the aneurysm wall is associated with functional abnormalities of ECs. These enzymes can degrade the extracellular matrix (ECM), leading to further thinning of the vascular wall and the formation of aneurysms [7]. Therefore, BMVECs may become a critical target for CA treatments.

The vascular endothelial growth factor (VEGF) is a critical signaling protein that promotes angiogenesis and vascular permeability. It plays a key role in both physiological and pathological processes, including embryonic development, wound healing, and tumor growth [8, 9]. VEGF‐A, one of the main isoforms, is particularly important for stimulating new blood vessel formation and maintaining vascular integrity. In the context of cerebral aneurysms, abnormal VEGF‐A expression has been implicated in promoting angiogenesis and vascular remodeling, contributing to the development and rupture of aneurysms [10]. Egr‐1 is a zinc‐finger transcription factor that regulates a wide range of cellular processes, including cell growth, differentiation, and inflammatory responses [11, 12]. It is rapidly induced by various stimuli, such as growth factors, cytokines, and stress signals. Egr‐1 can modulate the expression of multiple downstream genes, including those involved in inflammation and angiogenesis [13]. Recent studies have shown that Egr‐1 is a key regulator of blood–brain barrier damage induced by bacterial infection, and it can upregulate the expression of VEGF‐A, platelet‐derived growth factor subunit B (PDGFB), and angiopoietin‐like 4 (ANGPTL4), thereby enhancing vascular permeability and inflammation [14].

In 1996, Lee et al. screened for molecules that inhibit metastasis and identified KISS1 [15]. Kisspeptin (KP) is the product of KISS1, and its synthetic neurons are located in two main areas of the hypothalamus: the preoptic area and the arcuate nucleus. After C‐terminal amidation and proteolysis, KP produces peptides KP‐54, KP‐14, KP‐13, and KP‐10, all of which have the same 10‐amino‐acid amidated sequence at the C‐terminus, belong to the RF amide peptide family, and possess biological activity [16, 17]. These peptides attach to the G‐protein coupled receptor GPR54, a component of the GPCR family [18]. KP‐10 boosts the attachment of human monocytes to human umbilical vein ECs (HUVECs) and intensifies the creation of foam cells in human monocyte‐derived macrophages. In ApoE^−/−^ mice, KP‐10 hastens the progression of aortic atherosclerotic plaques [19]. However, the role of KP‐10 in CAs has been less reported. This study aimed to investigate whether the KP‐10/GPR54 system, a neuroendocrine pathway primarily involved in reproductive and metabolic regulation, plays a protective role in CA pathogenesis by modulating Egr‐1‐mediated MMP‐9/VEGF‐A signaling, macrophage infiltration, and angiogenesis.

## 2 Materials and Methods

### 2.1 Evaluation of Serum KP‐10 Levels in CA Patients Compared to Healthy Individuals

Ten pairs of CA patients and healthy subjects were included in our hospital, and informed consents were obtained. Blood from each subject was collected, which was centrifuged for obtaining the serum. KP‐10 levels in serum were determined using the ELISA method with commercial kits (KBH3919, Krishgen, China), with protocols strictly followed.

### 2.2 CA Modeling and Treatments

Sixty GPR54 KO mice (12 per group) with the background of C57BL/6 were obtained from Jackson laboratory. KP‐10 (50 μL, 1 mM, Cat#abs45153268, Absin, China) was injected into the peritoneal cavity of WT and GPR54 KO mice daily for 1 week prior to the onset of CA induction, and this regimen was maintained throughout the study duration. In contrast, mice in the WT and GPR54 KO groups received an equivalent measure of standard saline solution as a control. For CA modeling, rodents were rendered unconscious by 40 mg/kg pentobarbital sodium (P3761, Sigma‐Aldrich, United States), delivered via intraperitoneal injection. Following abdominal incision, renal arteries were identified, and silk sutures were used to secure them, employing 8–0 nylon threads. Additionally, the left common carotid artery was isolated and tied off. The surgical opening was then closed with sutures and sanitized, and the rodents were permitted to recuperate. Postoperatively, at the 2‐week mark, an enzyme solution of elastase (10 μL, 1 unit/mL in PBS, Worthington Biochemical Corporation, United States) was introduced into the right basal cistern utilizing a stereotaxic apparatus equipped with a mouse adapter (Stoelting, United States). The specific injection coordinates were 1.2 mm in front of the bregma, 0.7 mm away from the midline, and extending 5.0 mm beneath the cerebral cortex. Subsequently, rodents were furnished with a subdermal osmotic minipump (Durect, United States) for the ongoing delivery of angiotensin II (BACHEM, United States) dissolved in PBS (1000 ng/kg/min). Following the elastase administration and the insertion of the osmotic pump, rodents were placed on a dietary regimen that included 8% sodium chloride and 0.12% β‐aminopropionitrile.

### 2.3 Measurement of Blood Pressure in Mice

Systolic blood pressure was determined utilizing the tail‐cuff technique with the BP‐2000 Blood Pressure Monitoring System (Visitech Systems, United States) at 7 weeks after the surgery.

### 2.4 Verhoeff‐Van Gieson Staining

After repairing the COW specimen in formalin for a day, the specimen was rinsed under running tap water for 4 h. Subsequently, it was soaked in a series of alcohol solutions with increasing concentration, followed by drying with pure ethanol and xylene, and then encased in paraffin. Slices were made to a depth of 5 μm. The specimen sections were baked for 90 min to remove the wax. Verhoeff's solution was used for staining for 60 min, after which sections were placed in a 2% FeCl_3_ solution for 1–2 min. Then, sections were rinsed in a 5% thiosemicarbazide solution for 5 min, followed by soaking in VG stain for 3–5 min. Ultimately, sections were dried using a sequence of ethanol mixtures with escalating potency (70%, 90%, 95%, and 100% ethanol) and clarified with xylene. They were finally sealed with neutral adhesive and examined using a microscope (Mcalon, China). The dimension of the aneurysm was determined by averaging the largest vertical and horizontal measurements.

### 2.5 Immunohistochemistry

Paraffin sections were placed in an oven at 60°C for 1 h, then soaked in xylene I and xylene II for 15 min each, followed by sequential immersion in different amounts of ethanol and water for 5 min per time to complete the deparaffinization process. After deparaffinization, the paraffin sections were immersed in antigen retrieval solution (sodium citrate buffer, pH 6.0) and subjected to retrieval in a water bath at 95°C for 6 min. After rinsing with PBS and blocking, they were incubated with primary antibody GPR54 (1:200, Cat#ab137483, Abcam, United States) or CD36 (1:200, Cat#ab252922, Abcam, United States) at 4°C overnight, followed by incubation with biotin‐labeled secondary antibody (1:400, ab198657, Abcam, United States) at room temperature for 2 h. Then, they were incubated in chromogenic solution A + B (1:1) for 1 h and stained with 3,3′‐diaminobenzidine (DAB) for 5–10 min. After timely termination, they were dehydrated with gradient concentrations of ethanol and treated with xylene before being sealed with neutral gum and observed and photographed under a microscope.

### 2.6 Cell Culture, Transduction, and Treatment

HBMVECs were sourced from American Type Culture Collection (ATCC) (United States) and propagated in endothelial growth medium (EGM) enriched with 10% FBS, under an atmosphere of 5% CO_2_ and at a temperature of 37°C. To achieve Egr‐1 overexpressed HBMVECs, cells were transduced with lentivirus containing an Egr‐1‐overexpressed vector (lentiviral Egr‐1) for 2 days. To silence GPR54 in HBMVECs, cells were infected by a lentiviral vector carrying a short hairpin RNA aimed at GPR54 (lentiviral Gpr54 shRNA) for 2 days. Western blot analysis was utilized to confirm transfection efficacy in HBMVECs.

### 2.7 Real Time Polymerase Chain Reaction (PCR)

Total RNA from HBMVECs or COW area of mice was collected according to the guidelines included with the RNA isolation kit (NovoBiotec, China). The quantity of extracted RNA was verified using a miniaturized protein assay instrument. The RNA was converted into cDNA employing a reverse transcription system (Qiagen, Germany), and the synthesized cDNA was diluted to a volume of 50 μL for use in qRT‐PCR assays. The qRT‐PCR was assembled according to the protocol of the SYBR Green master mix kit (Applied Biosystems, United States), and the mixture was aliquoted into a specialized 384‐well plate designed for fluorescence detection to initiate the reaction. Glyceraldehyde‐3‐phosphate dehydrogenase (GAPDH) was used as a housekeeping gene. Gene expression levels were determined based on the cycle threshold (Ct) values using the 2^−ΔΔCt^ comparative quantification method obtained from the analysis.

### 2.8 Western Blot Analysis

The overall protein content from HBMVECs was isolated utilizing radioimmunoprecipitation assay (RIPA) disruption buffer. Once the concentration of proteins was quantified, a 30 μg sample was selected and combined with an adequate volume of loading buffer. This mixture underwent gel electrophoresis on a 10% SDS‐polyacrylamide gel (SDS‐PAGE), and the separated proteins were then transferred onto a nitrocellulose membrane with the aid of a water‐based transfer device. The membrane was subsequently incubated with a blocking agent at 4°C for a duration of 2 h. Specific primary antibodies targeting Egr‐1 (1:800, ab182624, Abcam, United States), Gpr54 (1:800, Cat#ab100896, Abcam, United States), and β‐actin (1:2000, Cat#ab137483, Abcam, United States) were introduced and allowed to react overnight at 4°C. Thereafter, secondary antibodies (1:2000, Cat#ab288151, Abcam, United States) were added, and the incubation continued at 37°C for 2 h. The membrane was then treated with an ECL detection reagent for visualization, and the resulting image was processed and analyzed using the Image J software for image analysis.

### 2.9 Enzyme‐Linked Immunosorbent Assay

COW tissues were homogenized and centrifuged to retrieve the supernatant fluid for analysis. Blood from mice was collected, which was centrifuged to obtain the serum as samples. The supernatant from HBMVECs was acquired by centrifugation for sample collection. KP‐10 levels in mouse serum were tested utilizing the ELISA kit (Cat#KBH3919, Krishgen, China). MMP‐9 (Cat#ml037717, Mlbio, China) and VEGF‐A (Cat#ml037273, Mlbio, China) levels in COW tissues were checked by commercial ELISA kits, respectively. Moreover, MMP‐9 (Cat#ml058617, Mlbio, China) and VEGF‐A (Cat#ml060752, Mlbio, China) levels in HBMVECs were determined by commercial ELISA kits, respectively. Briefly, the ELISA plates were coated with a capture antibody specific to the target protein and incubated overnight at 4°C. Following coating, the plates were blocked with 5% bovine serum albumin (BSA) for 1 h at room temperature. Subsequently, serum samples or supernatants were added to the coated wells and incubated for 90 min at 37°C. An enzyme‐linked detection antibody was then added and incubated for 1 h at room temperature. Finally, the substrate 3,3′,5,5′‐tetramethylbenzidine (TMB) was added to the wells, and the color change was measured using a plate reader at 450 nm. A standard curve was generated using serial dilutions of KP‐10 standards to quantify the concentration of protein in the samples.

### 2.10 Angiogenic Tubes Formation

A 96‐well plate was taken and coated with 50 μL of prepared mouse tail collagen per well, which was then solidified at 37°C for 1 h. HBMVECs were digested with trypsin and seeded into the pre‐coated mouse tail collagen culture plate at a density of 1.5 × 10^4^ cells per well. After a 12‐h incubation, the old culture medium was discarded, and HBMVECs were cultured for another 12 h with EGM containing 2% FBS to synchronize the cells. An additional layer of prepared mouse tail collagen was added on top, and the culture was continued for another 12 h. The formation of tubules was observed under an inverted microscope (ZEISS, Germany, ×100), and tubule formation was calculated by randomly selecting 5 fields of view per well and taking the average value.

### 2.11 Statistical Analysis

Experimental data were analyzed using SPSS version 10.0. The normality of continuous variables was assessed using the Kolmogorov–Smirnov test, and homogeneity of variance was evaluated with Levene's test. Data with a normal distribution (mean ± SD) were analyzed using a *t*‐test for comparisons between two groups and analysis of variance (ANOVA), followed by Scheffé's test for multiple group comparisons. For data that did not follow a normal distribution, the Kruskal–Wallis test was used for multiple group comparisons, followed by post hoc Dunn's test. A *p*‐value of < 0.05 was considered statistically significant.

## 3 Results

### 3.1 The KP‐10/GPR54 Signaling Pathway Is Dysregulated in Both CA Patients and Animal Models

**FIGURE 1:**
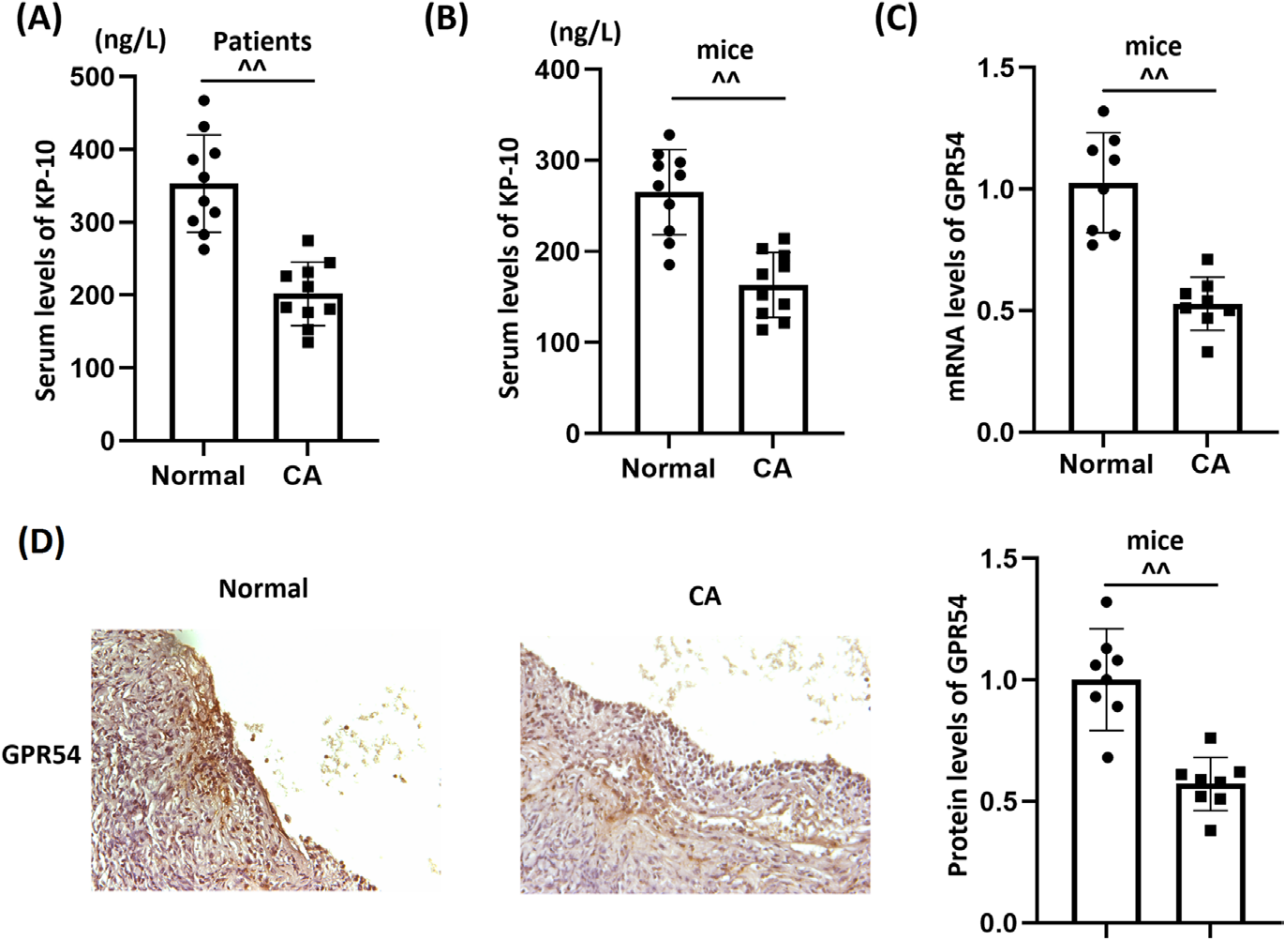
The Kisspeptin‐10 (KP‐10)/GPR54 signaling is dysregulated in both individuals with cerebral aneurysms (CA) and in mice with experimentally induced CA. (A) The serum levels of KP‐10 were measured in both control subjects and patients with CA using an ELISA method; (B) the serum levels of KP‐10 in both control and CA mice were determined using an ELISA; (C) mRNA levels of GPR54 in the COW region of both control and CA mice; (D) protein levels of GPR54 in the COW region of both control and CA mice as measured by immunohistochemistry (^^*p* < 0.01 vs. Normal group).

Firstly, in CA patients, serum KP‐10 levels were significantly reduced from 353.2 to 201.8 ng/L (Figure 1A). Similarly, serum KP‐10 levels in CA mice were substantially decreased from 265.1 to 163.3 ng/mL (Figure 1B). Moreover, GPR54 expression was markedly suppressed in the COW regions of CA animals (Figure 1C,D). Activating the KP‐10/GPR54 pathway may therefore be beneficial for the treatment of CA.

### 3.2 KP‐10 Administration Prevents CA Formation in WT Mice, but Not in GPR54 KO Mice

**FIGURE 2:**
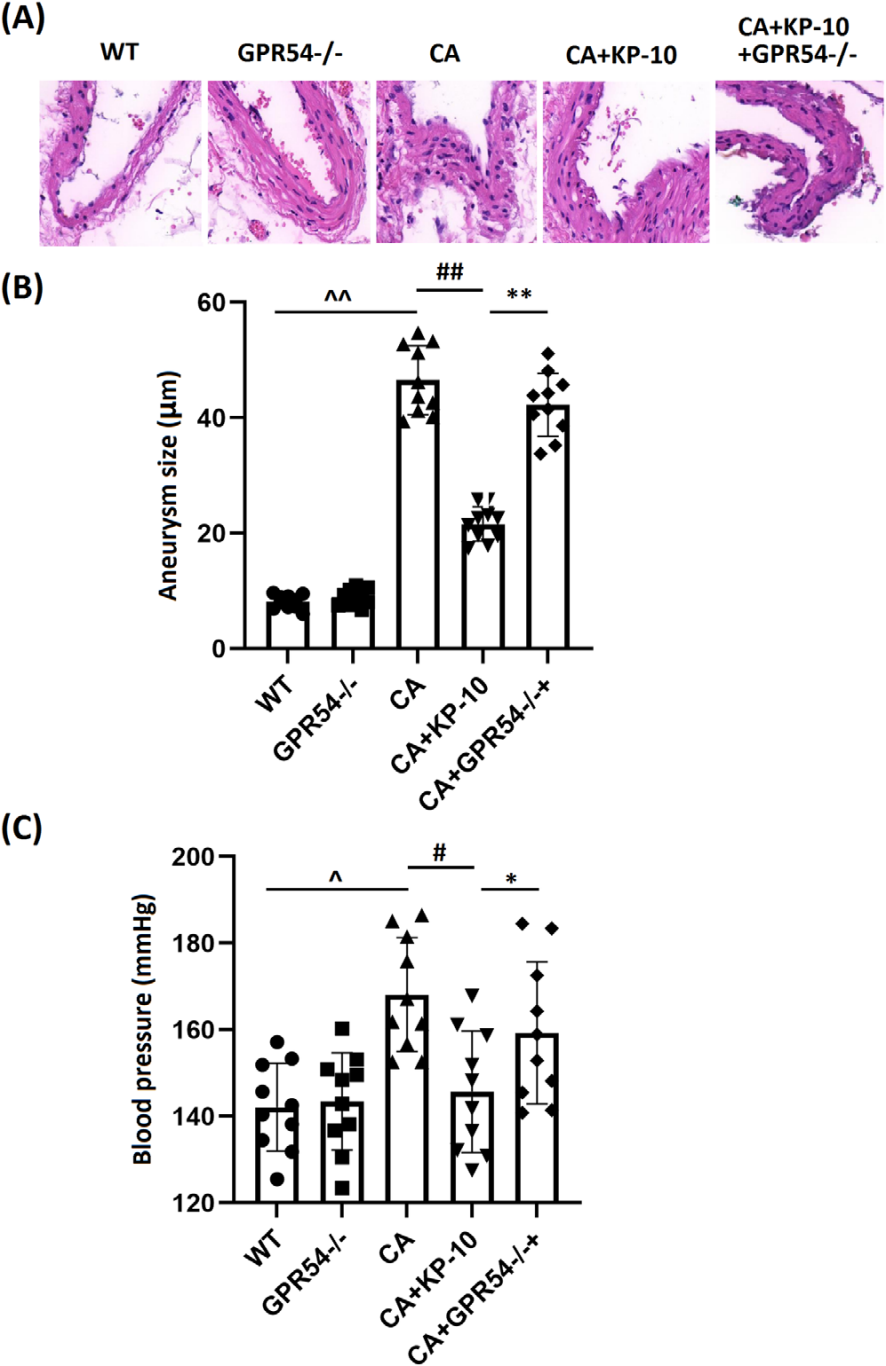
Administration of KP‐10 prevents CA formation in wild type but not GPR54 KO mice. Mice were divided into five groups: WT, GPR54^−/−^, CA, CA + KP‐10, and CA + GPR54^−/−^ + KP‐10. (A) Representative images of Verhoeff‐van Gieson staining of the vascular wall; (B) measurement of aneurysm size; (C) systolic blood pressure in different groups of mice at 7‐week after the surgery (^^,^^^ *p* < 0.05, 0.01 vs. WT group; ^#,##^ *p* < 0.05, 0.01 vs. CA group; *,**p < 0.05, 0.01 vs. CA + KP10 group).

To evaluate the therapeutic efficacy of KP‐10 in CA, WT mice and GPR54 KO mice were administered KP‐10. The rodents were categorized into five groups: WT, GPR54^−/−^, CA, CA + KP‐10, and CA + GPR54^−/−^ + KP‐10. Representative images of Verhoeff‐van Gieson staining for vascular walls are shown in Figure 2A. The aneurysm size in the WT and GPR54^−/−^ groups was 8.1 and 8.9 μm, respectively, while it was significantly increased to 46.5 μm in CA mice. This increase was strikingly reduced to 21.6 μm by KP‐10 treatment. However, compared to KP‐10‐treated WT mice, the aneurysm size in KP‐10‐treated GPR54^−/−^ mice was notably reversed to 42.3 μm (Figure 2B). Additionally, systolic blood pressures at 7 weeks post‐surgery in the WT, GPR54^−/−^, CA, CA + KP‐10, and CA + GPR54^−/−^ + KP‐10 groups were 142.1 mmHg, 143.4 mmHg, 168.1 mmHg, 145.7 mmHg, and 159.3 mmHg, respectively (Figure 2C).

### 3.3 KP‐10 Represses MMP‐9 Levels in the COW Region of CA Mice

**FIGURE 3:**
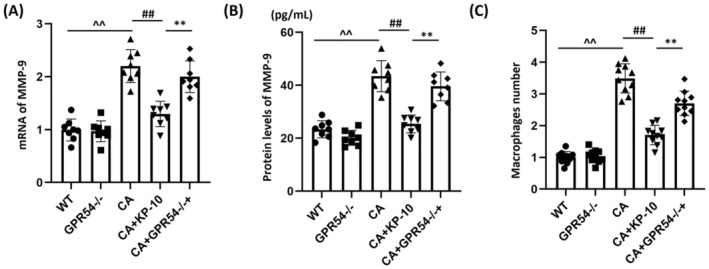
Administration of KP‐10 inhibits the expression of MMP‐9 in the COW region of CA mice. Mice were divided into five groups: WT, GPR54^−/−^, CA, CA + KP‐10, and CA + GPR54^−/−^ + KP‐10. (A) mRNA of MMP‐9; (B) protein levels of MMP‐9 as measured by ELISA; (C) the infiltrated macrophages were staining with CD36 and the number was counted (^^^^ *p* < 0.01 vs. WT group; ^##^ *p* < 0.01 vs. CA group; **p < 0.01 vs. CA + KP10 group).

MMP‐9 expression was slightly altered in the GPR54^−/−^ group and was significantly enhanced in CA mice, but this increase was notably repressed by KP‐10 treatment. Compared to KP‐10‐treated WT mice, MMP‐9 expression in KP‐10‐treated GPR54^−/−^ mice was considerably increased (Figure 3A). Consistent with PCR results, MMP‐9 protein levels in the WT, GPR54^−/−^, CA, CA + KP‐10, and CA + GPR54^−/−^ + KP‐10 groups were 23.4, 20.16, 43.5, 25.1, and 39.6 pg/mL, respectively (Figure 3B). Additionally, the number of CD36‐positive cells in COW tissues was mildly altered in the GPR54^−/−^ group and was noticeably enhanced in CA mice, but this enhancement was significantly reduced by KP‐10 treatment. Compared to KP‐10‐treated WT mice, the number of CD36‐positive cells in COW tissues was markedly increased in KP‐10‐treated GPR54^−/−^ mice (Figure 3C). These results suggest that the effects of KP‐10 may depend on GPR54.

### 3.4 KP‐10 Downregulates VEGF‐A in the COW Region of CA Mice

**FIGURE 4:**
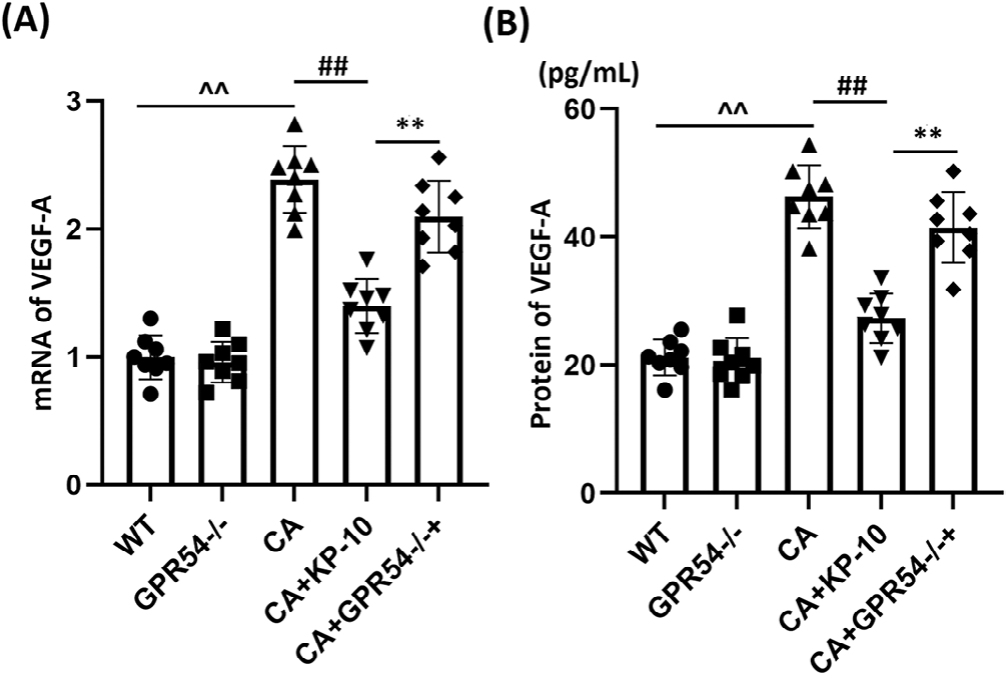
Administration of KP‐10 inhibits the expression of VEGF‐A in the COW region. (A) mRNA levels of VEGF‐A; (B) the protein levels of VEGF‐A as measured by ELISA (^^^^ *p* < 0.01 vs. WT group; ^##^ *p* < 0.01 vs. CA group; **p < 0.01 vs. CA + KP10 group).

VEGF‐A is involved in the abnormal angiogenesis that occurs during CA [20]. Here, gene expression of VEGF‐A was mildly altered in the GPR54^−/−^ group and was significantly enhanced in CA mice, which was notably repressed by KP‐10. Compared to KP‐10‐treated WT mice, VEGF‐A expression in KP‐10‐treated GPR54^−/−^ mice was notably elevated (Figure 4A). Consistent with PCR results, VEGF‐A protein levels in the WT, GPR54^−/−^, CA, CA + KP‐10, and CA + GPR54^−/−^ + KP‐10 groups were 21.2, 20.55, 46.32, 27.3, and 41.5 pg/mL, respectively (Figure 4B). These findings suggest that the beneficial effects of KP‐10 were abrogated in GPR54^−/−^ mice.

### 3.5 KP‐10 Ameliorates Ang II‐Induced Cell Proliferation and Tube Formation in HBMVECs

**FIGURE 5:**
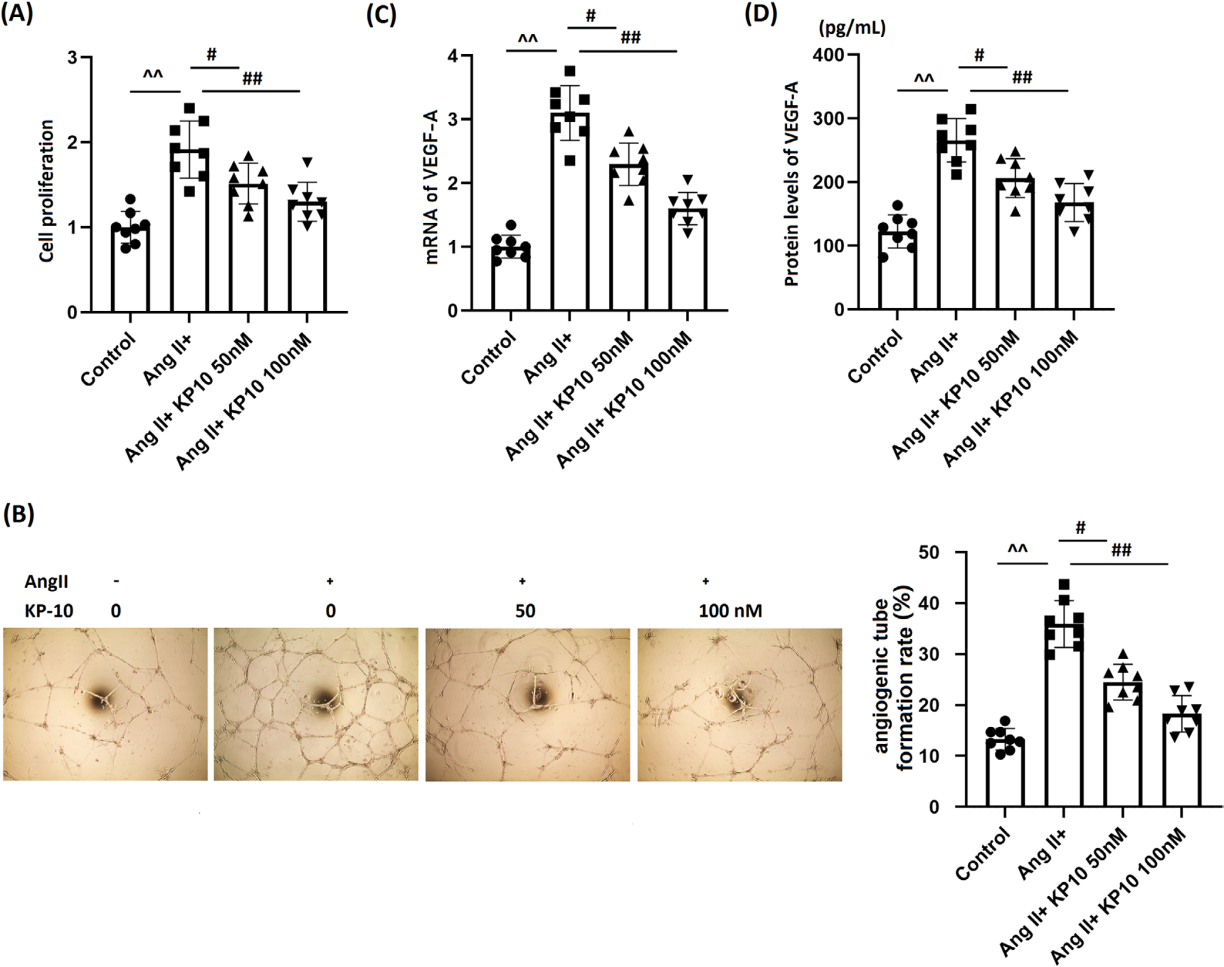
Treatment with KP‐10 ameliorates Ang II‐ induced cell proliferation and angiogenic tube formation of human BMVECs (HBMVECs). Cells were stimulated with Ang II (10^−7^ mol/L) with or without KP‐10 (50, 100 nM). (A) Cell proliferation was measured by CCK‐8; (B) angiogenic tube formation rate; (C) mRNA of VEGF‐A; (D) protein levels of VEGF‐A (^^^^ *p* < 0.01 vs. Control group; ^#,##^ *p* < 0.05, 0.01 vs. Ang II group).

HBMVECs were stimulated with Ang II (10^−7^ mol/L) with or without KP‐10 (50, 100 nM). The cell viability of HBMVECs was significantly enhanced by Ang II, which was markedly restrained by 50 and 100 nM KP‐10 (Figure 5A). Additionally, the tube formation rate of Ang II‐challenged HBMVECs was enhanced from 13.4% to 35.9%, which was significantly reduced to 24.5% and 18.3% by 50 and 100 nM KP‐10, respectively (Figure 5B). VEGF‐A expression was sharply enhanced in Ang II‐challenged HBMVECs but was notably decreased by 50 and 100 nM KP‐10 (Figure 5C). Moreover, VEGF‐A levels in the control, Ang II, 50 nM KP‐10, and 100 nM KP‐10 groups were 122.6, 265.5, 203.6, and 165.5 pg/mL, respectively (Figure 5D).

### 3.6 The Protective Effects of KP‐10 Are Mediated by Egr‐1 in HBMVECs

**FIGURE 6:**
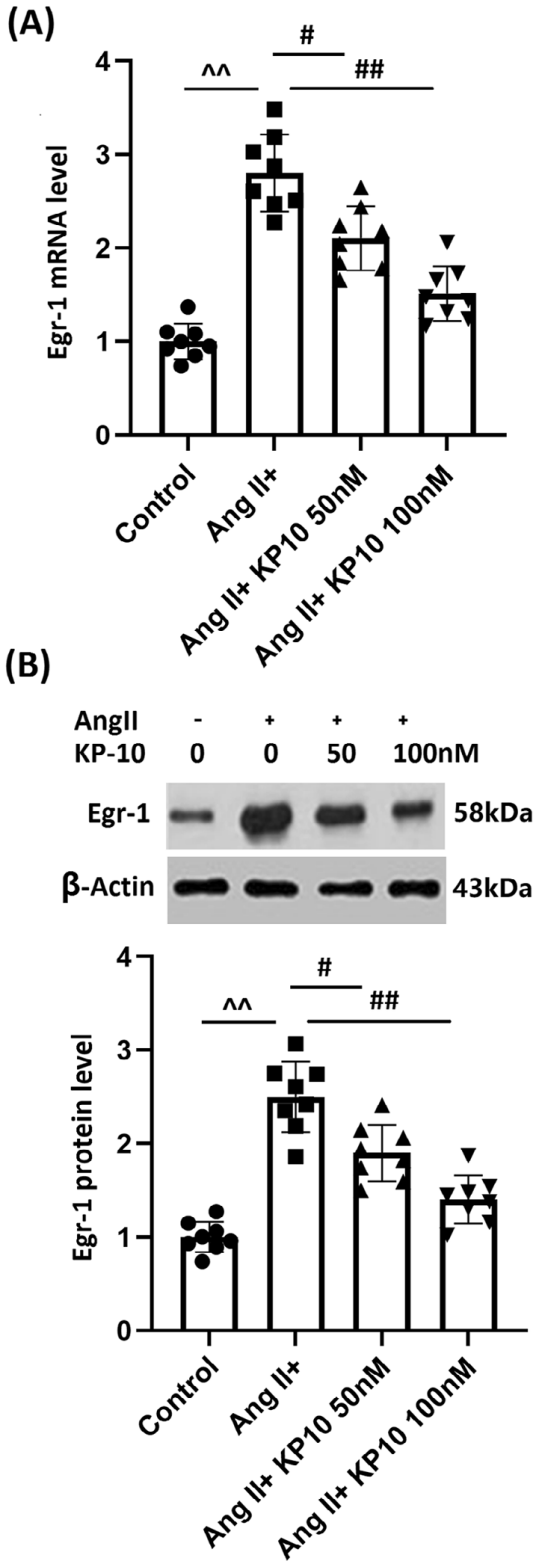
Treatment with KP‐10 inhibits Ang II‐ induced expression of Egr‐1 in HBMVECs. Cells were stimulated with Ang II (10^−7^ mol/L) with or without KP‐10 (50, 100 nM). (A) mRNA of Egr‐1; (B) protein of Egr‐1 (^^^^ *p* < 0.01 vs. Control group; ^#,##^ *p* < 0.05, 0.01 vs. Ang II group).

**FIGURE 7:**
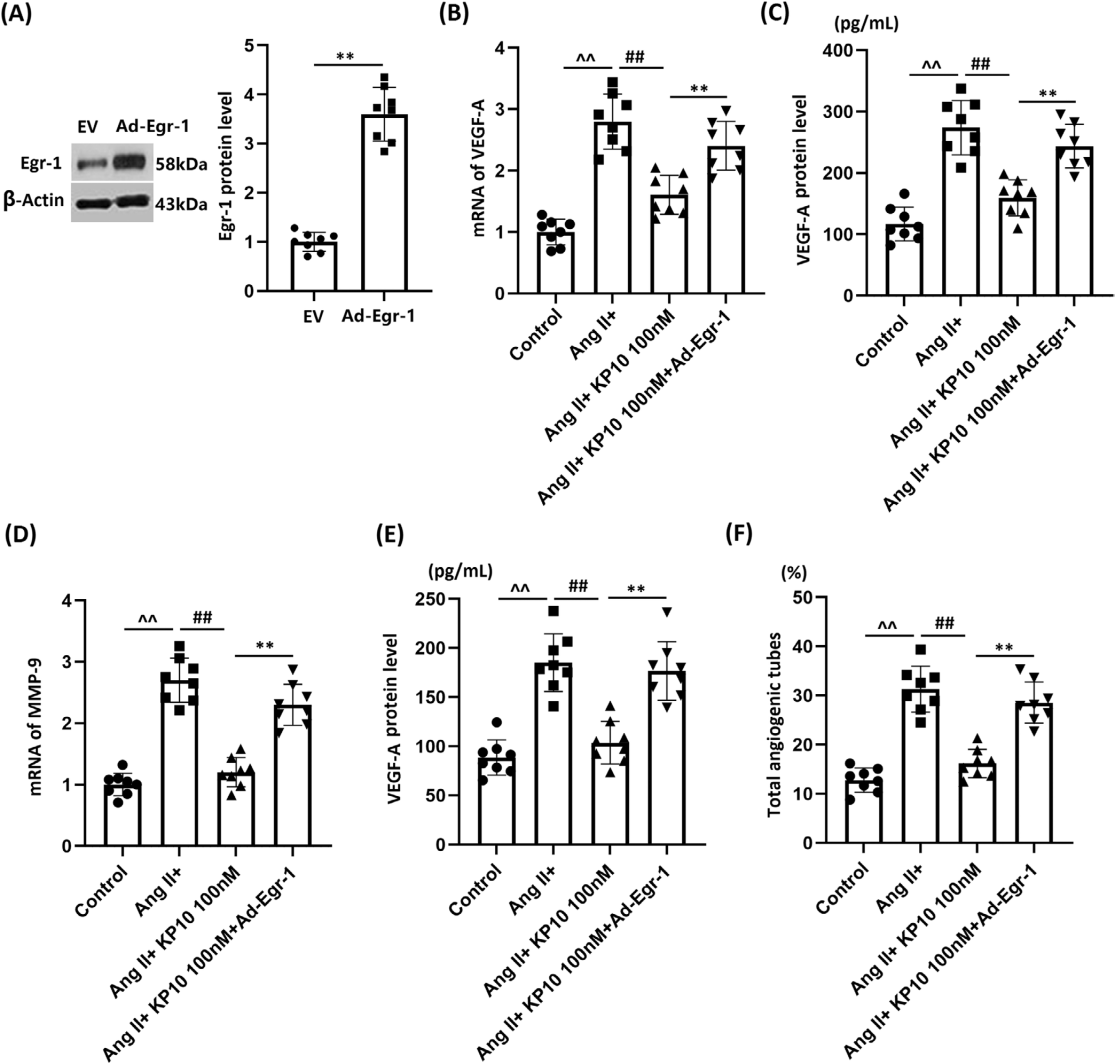
Overexpression of Egr‐1 abolishes the beneficial effects of KP‐10 in inhibiting the expression of VEGF‐A, MMP‐9, and tube formation of HBMVECs. Cells were transduced with lentiviral Egr‐1, followed by stimulation with Ang II (10^−7^ mol/L) with or without KP‐10 (100 nM). (A) Western blot results revealed successful overexpression of Egr‐1; (B) mRNA of VEGF‐A; (C) protein of VEGF‐A; (D) mRNA of MMP‐9; (E) protein levels of MMP‐9; (F) total angiogenic tubes of HBMVECs (^^^^ *p* < 0.01 vs. Control group; ^##^ *p* < 0.01 vs. Ang II group; **p < 0.01 vs. Ang II + KP‐10 group).

Egr‐1 is a transcription factor reported to mediate VEGF‐A expression [21]. Here, we found that Egr‐1 levels were significantly enhanced in Ang II‐stimulated HBMVECs but were sharply restrained by 50 and 100 nM KP‐10 (Figure 6A,B). HBMVECs were transduced with lentiviral Egr‐1, followed by stimulation with Ang II (10^−7^ mol/L) with or without KP‐10 (100 nM). Egr‐1 overexpression in HBMVECs was validated by Western blot analysis (Figure 7A). VEGF‐A gene and protein levels were sharply enhanced in HBMVECs by Ang II, which were noticeably repressed by KP‐10. After Egr‐1 overexpression, VEGF‐A gene and protein levels were strikingly reversed (Figure 7B,C). Upregulated MMP‐9 levels in Ang II‐challenged HBMVECs were largely repressed by KP‐10 but were notably reversed by Egr‐1 overexpression (Figure 7D). MMP‐9 production in HBMVECs was enhanced from 88.6 to 185.2 pg/mL by Ang II, which was remarkably reduced to 103.7 pg/mL by KP‐10. Following Egr‐1 overexpression, MMP‐9 production was reversed to 176.8 pg/mL (Figure 7E). Moreover, the tube formation rate in the control, Ang II, Ang II + KP‐10, and Ang II + KP‐10+ lentiviral Egr‐1 groups was 12.8%, 31.3%, 16.2%, and 28.6%, respectively (Figure 7F).

### 3.7 Silencing Gpr54 Counteracts the Effects of KP‐10 in Inhibiting Egr‐1 in HBMVECs

**FIGURE 8:**
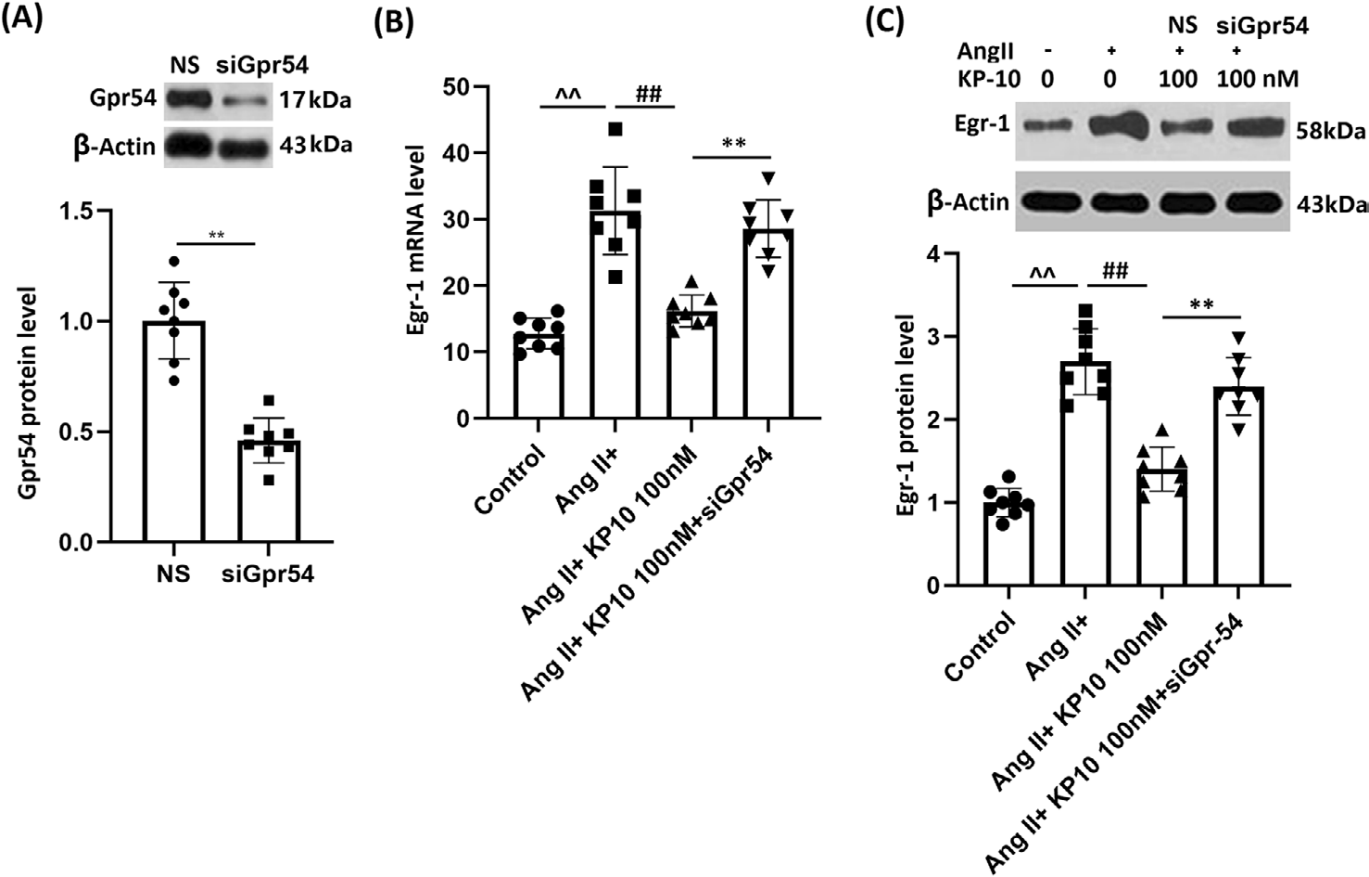
Silencing of Gpr54 counteracts the effects of KP‐10 in inhibiting the expression of Egr‐1 in HBMVECs. Cells were transduced with lentiviral Gpr54 shRNA, followed by stimulation with Ang II (10^−7^ mol/L) with or without KP‐10 (100 nM). (A) Western blot analysis revealed successful silencing of Gpr54; (B) mRNA of Egr‐1; (C) protein of Egr‐1 (^^^^ *p* < 0.01 vs. Control group; ^##^ *p* < 0.01 vs. Ang II group; **p < 0.01 vs. Ang II + KP‐10 group).

Cells were transduced with lentiviral Gpr54 shRNA, followed by Ang II stimulation (10^−7^ mol/L) with or without KP‐10 (100 nM). GPR54 knockdown in HBMVECs was validated by Western blot analysis (Figure 8A). Markedly enhanced Egr‐1 levels in Ang II‐challenged HBMVECs were repressed by KP‐10, but were strikingly increased by silencing GPR54 (Figure 8B,C).

## 4 Discussion

The formation of CA is related to a variety of factors, including hemodynamics, the bioactivity of the vascular wall, and genetic and epidemiological factors [22]. ECs fulfill a crucial function in the formation of aneurysms by not only responding to changes in blood flow but also participating in inflammatory reactions and the remodeling of the ECM [23]. During CA formation, angiogenesis supports the growth of aneurysms by providing nutrients and oxygen, and also promotes the rupture of aneurysms by increasing the fragility of blood vessels [24]. Studies indicate that signaling pathways related to angiogenesis are abnormally enhanced in CA ECs, and specific genes such as Angiopoietin‐2 (ANGPT2) participate in aneurysm formation [7]. In addition, inflammatory factors such as C‐C motif chemokine ligand 21 (CCL21) may also promote angiogenesis and the migration of inflammatory cells in the formation of aneurysms [25]. Angiogenesis involves a variety of cell types and signaling pathways, including ECs, pericytes, and their interactions with the ECM [26]. The KP family, encoded by the KiSS‐1 gene, includes several peptides such as KP‐54, KP‐14, KP‐13, and KP‐10, all of which act as endogenous ligands for the GPR54 receptor [27]. While our study focuses on the role of KP‐10 in CAs, other KP family members have also been implicated in various physiological and pathological processes. KP‐54 is the longest isoform of the KP family. It has been shown to have significant effects on neuronal function and motor performance. For example, chronic intranasal administration of KP‐54 has been found to mitigate motor deficits and protect dopaminergic neurons in the nigrostriatal pathway [28]. In the context of brain vascular diseases, KP‐54 may have similar vasoactive properties as KP‐10, potentially influencing vascular tone and permeability. KP‐14 and KP‐13 are shorter isoforms that also exhibit vasoconstrictive properties, similar to KP‐10, and have been shown to be potent vasoconstrictors in isolated coronary artery rings [29]. Their roles in brain vascular diseases are less studied, but given their vasoactive properties, they may contribute to vascular remodeling and inflammation, processes that are critical in the development of CAs and other cerebrovascular disorders. Given the vasoactive and inflammatory modulatory properties of KP family members, it is plausible that they could play roles in the pathogenesis of CAs and other brain vascular diseases. For instance, KP‐10 has been shown to accelerate atherosclerotic plaque progression and instability, leading to plaque rupture [19]. Similar mechanisms may be at play with other KP family members, although further research is needed to elucidate their specific contributions.

Here, KP‐10/GPR54 signaling was remarkably inactivated in both CA patients and CA mice, hinting that activating KP‐10/GPR54 signaling might bring benefits for treating CA. Parallel to results claimed by Chen [30], prolonged aneurysm size and enhanced systolic blood pressure were noted in CA mice, which were strikingly mitigated by KP‐10, hinting at an anti‐CA property of KP‐10 in a mouse model. However, in GPR54 KO mice, the anti‐CA property of KP‐10 was not observed, indicating that KP‐10 exerted anti‐CA effects via activating GPR54. Referencing Yi's method [31], Ang‐II was utilized for establishing in vitro models in ECs for simulating aneurysm. Activated proliferation and enhanced tube formation ability were observed in Ang‐II‐managed ECs, which were repressed by KP‐10, hinting that the anti‐CA property of KP‐10 might be connected with its inhibition against angiogenesis.

Stem cell‐derived monocytes exhibit responsiveness to the induction of cytokines, moving towards areas of blood vessel damage, penetrating the vascular lining, and maturing into macrophages [32, 33]. These macrophages actively produce a variety of cytokines to engage in inflammatory processes by modulating additional immune cells such as T lymphocytes and mast cells [34]. Moreover, macrophages release varying quantities of MMPs and their natural inhibitors (TIMPs) at various stages, causing a disruption in the equilibrium of MMPs and TIMPs within aneurysmal tissues, which subsequently results in the breakdown of vascular gelatin and the degradation of the ECM [35]. Harmonious with Yu's report [30], upregulated MMP‐9 and macrophage infiltration were noted in CA mice, which were noticeably repressed by KP‐10. However, no effects of KP‐10 on MMP‐9 expression and macrophage infiltration were observed in GPR54 KO CA mice, implying that KP‐10 exerted anti‐CA property via repressing macrophage infiltration by activating GPR54.

VEGF‐A is a crucial angiogenic factor that serves a vital purpose in angiogenesis. By binding to its receptor VEGFR‐2, VEGF‐A facilitates the proliferation, migration, and survival of ECs, thereby inducing the formation of new blood vessels [36, 37]. VEGF‐A is engaged in the pathogenesis of various angiogenesis‐related diseases, including CA [23, 38]. Here, VEGF‐A was substantially upregulated in both CA mice and Ang‐II‐managed ECs, contents of which were remarkably repressed by KP‐10, hinting that KP‐10 inhibited angiogenesis via repressing VEGF‐A productions. Egr‐1 is a vital transcription factor that participating in regulating cellular processes such as growth, differentiation, development, and proliferation [39]. Egr‐1 possesses three reiterated zinc‐finger regions, enabling it to attach to particular DNA strands and modulate the transcription of designated genes [40]. The regulation of Egr‐1 on VEGF's transcriptional has been previously claimed. Here, enhanced expressions of Egr‐1 in Ang‐II‐managed HBMVECs were noticeably repressed by KP‐10, implying that KP‐10 might inhibit VEGF‐A productions via repressing Egr‐1. Additionally, influences of KP‐10 in Ang‐II‐managed HBMVECs were strikingly reversed by overexpressing Egr‐1, which validated that KP‐10 suppressed VEGF‐A‐mediated angiogenesis during CA via inhibiting Egr‐1. However, impacts of KP‐10 on Egr‐1's expressions in Ang‐II‐managed HBMVECs were abrogated by silencing GPR54, hinting that KP‐10 repressed Egr‐1's function via activating GPR54.

This study has several limitations. First, it was conducted solely in a mouse model, and the lack of clinical validation means that the efficacy of KP‐10 in humans remains uncertain. Secondly, while the study explores KP‐10's effects on cerebral aneurysms, its impact on different types of aneurysms has not been established. Additionally, the research concentrated exclusively on the KP‐10/GPR54 pathway and did not investigate other potential molecular mechanisms that may contribute to aneurysm development. Lastly, although KP‐10's effects are mediated through Egr‐1, its long‐term impact and potential side effects in various physiological or pathological conditions are still not fully understood.

In conclusion, our study elucidates the critical role of the KP‐10/GPR54 system in the pathogenesis of CAs. We demonstrate that KP‐10, acting via GPR54, exerts protective effects by inhibiting Egr‐1 expression, which subsequently suppresses the expression of MMP‐9 and VEGF‐A, reduces macrophage infiltration, and impedes angiogenesis. These findings highlight the KP‐10/GPR54 axis as a promising therapeutic target for the prevention and treatment of cerebral aneurysms. In future studies, the clinical trials of KP‐10 in treating CA will be applied to test its pharmacologic actions in CA patients.

## Ethics Statement

This study was approved by the Ethics Committee of the First Dongguan Affiliated Hospital of Guangdong Medical University (Approval No. NTC5638).

## Consent

All the authors agreed to publish this article.

## Conflicts of Interest

The authors declare no conflicts of interest.

## Data Availability

The data that support the findings of this study are available from the corresponding author upon reasonable request.
